# Host and Clostridioides difficile-Response Modulated by Micronutrients and Glutamine: An Overview

**DOI:** 10.3389/fnut.2022.849301

**Published:** 2022-06-20

**Authors:** Andréa V. Loureiro, Maria L. L. Barbosa, Maria L. G. S. Morais, Ismael P. Souza, Letícia S. Terceiro, Conceição S. Martins, Arkila P. R. Sousa, Renata F. C. Leitão, Jae H. Shin, Cirle A. Warren, Deiziane V. S. Costa, Gerly A. C. Brito

**Affiliations:** ^1^Department of Medical Sciences, Faculty of Medicine, Federal University of Ceará, Fortaleza, Brazil; ^2^Department of Morphology, Faculty of Medicine, Federal University of Ceará, Fortaleza, Brazil; ^3^Department of Pharmacology and Physiology, Faculty of Medicine, Federal University of Ceará, Fortaleza, Brazil; ^4^Division of Infectious Diseases and International Health, University of Virginia, Charlottesville, Virginia, VA, United States

**Keywords:** *Clostridium difficile*, calcium, selenium, iron, zinc, vitamin D, glutamine

## Abstract

Changes in intestinal microbiota are integral to development of *Clostridioides difficile* (*C. difficile*)—associated nosocomial diarrhea. Certain diets, especially Western diets, increase susceptibility to *C. difficile* infection (CDI). Here, we discuss recent findings regarding how nutrients modulate response of the host and *C. difficile* during infection. Calcium has a role in the sporulation and germination process. Selenium is effective in reducing the total amount of *C. difficile* toxin A (TcdA) and toxin B (TcdB) and in decreasing its cytotoxicity. In addition, selenium phosphate synthetase deficiency reduces *C. difficile* growth and spore production. On the other hand, iron has a dual role in *C. difficile* growth. For instance, high intracellular levels can generate reactive hydroxyl radicals, whereas low levels can reduce its growth. In humans, zinc deficiency appears to be related to the recurrence of CDI, in contrast, in the CDI model in mice a diet rich in zinc increased the toxin's activity. Low vitamin D levels contribute to *C. difficile* colonization, toxin production, and inflammation. Furthermore, glutamine appears to protect intestinal epithelial cells from the deleterious effects of TcdA and TcdB. In conclusion, nutrients play an important role in modulating host and pathogen response. However, further studies are needed to better understand the mechanisms and address some controversies.

## Introduction

*Clostridioides difficile* (*C. difficile*), a gram-positive, spore-forming, toxin-producing anaerobic bacterium, is a major cause of nosocomial diarrhea associated with antibiotic use (1, 2). Age (>65 years), female gender, comorbidities (such as chronic kidney disease, diabetes mellitus, leukemia, cancer, and inflammatory bowel disease), hospitalization, and immunosuppression are risk factors for acquiring *C. difficile* infection (CDI) (3). The incidence of CDI worldwide increased between 2001–2011, when it is stabilized (4). However, it remains a challenge in hospitals around the world and in the community. In European hospitals, seven cases of CDI occur for every 10,000 patients, costing ~$6,000 for each first case of CDI (5, 6). CDI is the tenth cause of 30-day readmission scans of gastrointestinal disease, and the fifth cause of death from non-malignant gastrointestinal disease in the U.S., costing ~$4 billion a year (5).

Changes in intestinal microbiota components is one of the key factors for CDI. Diets, especially Western, are often associated with changes in the intestinal microbiome (7). In an antibiotic-induced CDI rat model, the high-fat and high-protein diet increases the severity of CDI, whereas the high-carbohydrate diet protects (7). In another similar study, mice infected with *C. difficile* that consumed a dietary fiber with low microbiota accessible carbohydrates (MACs), perpetuated CDI after 12 days of infection (8). These diets may change the micronutrients availability into the gut by changing the microbiota, which in turn can contribute to CDI susceptibility. Micronutrients are required for many pathogenic cellular processes and are essential cofactors for a variety of proteins that are critical for bacterial life (9).

The transmission of *C. difficile* occurs *via* fecal-oral route or contact with the spores of this bacteria. The spores, after resisting gastric acid content, germinate in the small intestine, and then colonize the colon of the host, releasing several virulence factors, such as toxins A (TcdA) and B (TcdB) (10–12). The virulence factors act in the colon causing severe inflammation and mucosal disruption, resulting from mild diarrhea to pseudomembranous fulminate colitis (1).

To survive and cause disease, most bacterial pathogens need nutrients. Therefore, we performed a review of the literature on the role of specific nutrients such as calcium, selenium, iron, zinc, vitamin D, and glutamine in the pathogen and/or host response during CDI.

## Micronutrients

### Calcium

Calcium is an important micronutrient for the regulation of bacterial homeostasis. Its action is subdivided into several mechanisms that vary from the regulation of ion passage, which is important to the sporulation process, to protection against reactive oxygen species (ROS) (13). This mineral is known to be crucial in spore formation, a mechanism that allows bacteria to survive in various environments (14).

Kochan et al. (14) demonstrated that calcium present in the intestinal environment, in a glycine amino acid-dependent manner, facilitates spore germination of *C. difficile in vitro*. In mice with CDI, the calcium present in the bile is sufficient to induce sporulation, and intestinal calcium (present in the ileum region) at a concentration around 15 mM is responsible for *C. difficile* spore production. *C. difficile* spores can store large amounts of calcium on their outermost layers (<50 mM calcium), thus in the absence of intestinal calcium, there is activation of the calcium-dependent enzyme (in an intracellular form in the spore) that releases endogenous calcium into the extracellular medium, which combined with glycine, initiates the spore germination process. Thus, if this enzyme is inhibited the onset of germination is delayed (14).

In an *in vitro* study, TcdB treatment induced HeLa cells to release higher levels of calcium leading to increased activation of protein kinase C (PKC). Calcium has been shown to play an important role in TcdB-induced ROS synthesis in Hela cells, as blockade of L-type calcium channels decreased ROS production (15). Calcium binds to the L-type calcium channels, resulting in PKC activation (in the first 15 min after toxin challenge), which in turn activates the metabolites of nitric oxide, resulting in production of ROS (15). To our knowledge there are no reports about calcium uptake or serum calcium levels in CDI in patients or murine models.

### Selenium

Selenium is an essential micronutrient that plays a crucial role in the development of a variety of physiological processes (16). It is present in a wide variety of selenoproteins, including proline and glycine reductases, enzymes essential for carrying out Stickland metabolism. Stickland metabolism and its substrates play an important role in the pathogenesis of CDI (17).

Selenophosphate synthase (SelD) initiates the process of selenium incorporation into proteins through the generation of selenophosphate from hydrogen selenide. The absence of selenophosphate leads to decreased selenoprotein synthesis, which in turn causes changes in the physiology of *C. difficile* (17).

McAllister et al. (17) demonstrated that a strain of *C. difficile* deficient in SelD exhibits decreased growth and reduced spore production. However, its deficiency did not affect the early events of spore germination, but rather caused a delay in the growth of germinated spores. Furthermore, the authors proposed that the mutant strain was able to compensate for the loss of selenoprotein synthesis through upregulation of other metabolic pathways.

In contrast, Pellissery et al. (18) have found that selenium (sodium selenite) was effective in reducing TcdA and TcdB levels and in decreasing cytotoxicity of *C. difficile* culture supernatant on Vero cells. The authors have proposed that this effect on toxin production occurred due to the downregulation of genes important for toxin production (*tcdb, tcda*, and *tcdr*), as well as the up-regulation of *tcdc* (toxin negative regulator) and CodY (*tcdr* repressor). Moreover, selenium was also able to reduce the growth of *C. difficile* spores and decrease bacterial growth in the presence of ciprofloxacin (18).

It has been demonstrated that auranofin disrupts selenium metabolism in *C. difficile* by forming a stable Auranofin–Selenide complex (19). This complex formation caused the selenium deficiency for selenoenzymes synthesis and the consequent inhibition of *C. difficile* growth due to prevention of the uptake and nutritional utilization of Selenium (19).

Previous study reported that ebselen, a selenium-based organic complex, showed efficacy against autoproteolytic cleavage of both TcdA and TcdB, inhibiting toxin-mediated pathology *in vitro*, blocking lethal toxicity of TcdB in a systemic toxigenic mouse model, and was effective in a clinically relevant mouse model of CDI. The authors suggest that the protective effect of ebselen was mediated by its ability to block a cysteine protease domain (CPD), involved in the glucosyltransferase domain (GTD) processing (20).

**Figure 1:**
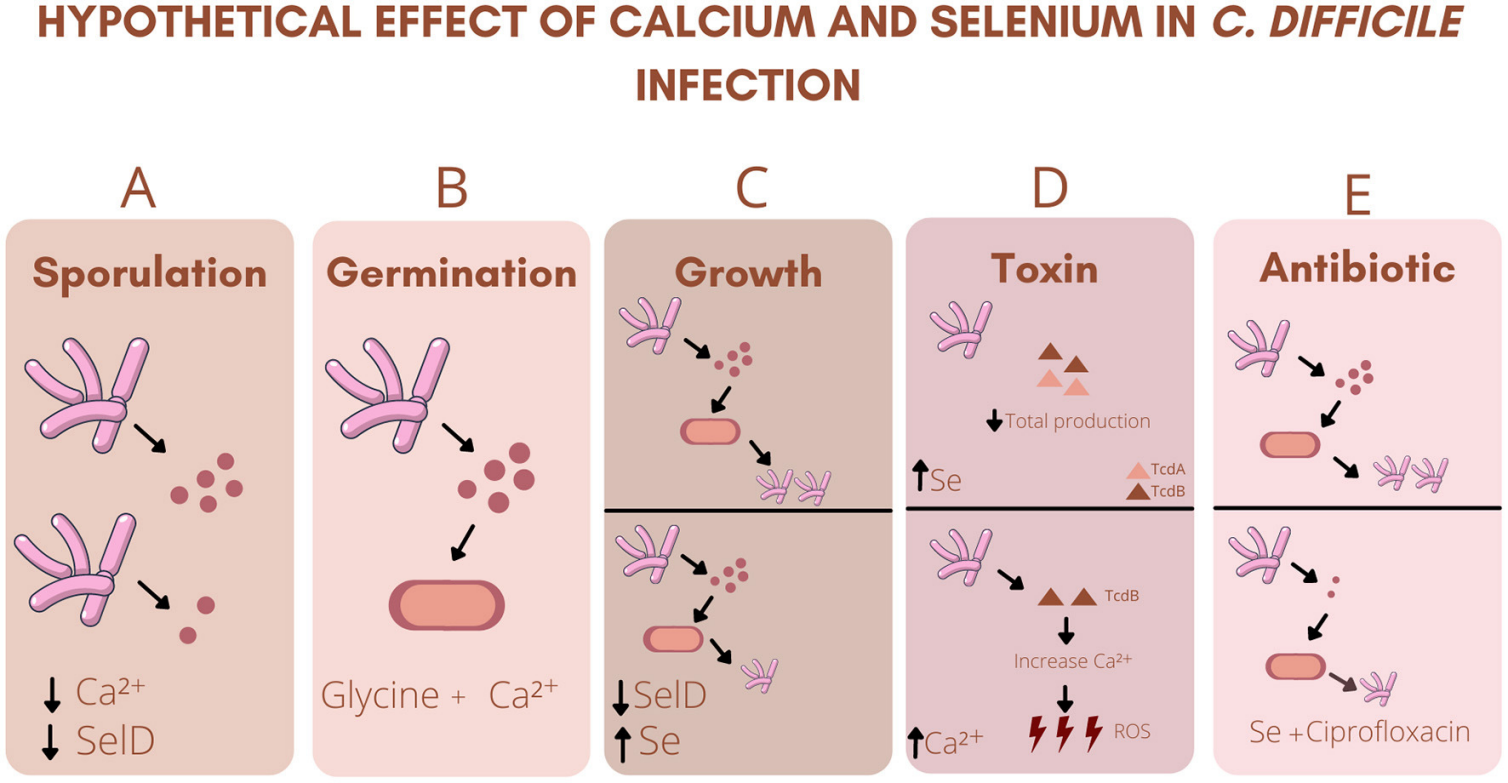
Hypothetical effect of calcium and selenium in *C. difficile* infection: **(A)** Decreased intestinal Ca^2+^ (Calcium) or SelD (Seleno phosphate synthetase) deficiency decreases spore formation. **(B)** Association between Ca^2+^ and Glycine is essential for the initiation of germination. **(C)** SelD deficiency decreased the growth of *C. difficile* and sodium selenite (Se) inhibited the growth of the strain. **(D)** High levels of Se decreases the total production of TcdA (*Clostridium difficile* toxin A) and TcdB (*Clostridium difficile* toxin B). High levels of TcdB increases Ca^2+^ levels, which in turn increases the production of ROS (Reactive oxygen species). **(E)** Association of Se with ciprofloxacin decreases the growth of *C. difficile*, as well as its sporulation.

Interestingly, sodium selenite facilitated the growth of beneficial bacteria to the intestinal microbiota (*Lactococcus lactis lacti, Lactobacillus rhamnosus, Lactobacillus delbrueckii bulgaricus, Lactobacillus reuteri, Lactobacillus brevis*, and *Lactobacillus plantarum*) (18). The normal intestinal microbiota plays a key role in protecting against CDI, as it provides colonization resistance against pathogenic bacteria (11). The imbalance in microbiota composition and the consequent reduction of beneficial bacteria species causes the breakdown of the integrity of the intestinal barrier and the loss of its functionality, making the individual more susceptible to CDI. A brief summary of the effects of calcium and selenium in CDI is shown in Figure 1.

### Iron

Iron is an essential cofactor for the survival of Gram-positive and Gram-negative bacteria (21). This nutrient has critical function for the strict anaerobe *C. difficile*. High intracellular levels of iron can generate reactive hydroxyl radicals, therefore maintenance of iron at the right levels is important (22). The ferric uptake regulator (Fur) is responsible for regulating the *C. difficile* response to iron limitation. The study carried out by Berges et al. (23) characterized the functional and metabolic alterations induced by Fur-mediated low iron response.

To investigate the role of Fur, the authors constructed a fur mutant *C. difficile* strain. Under iron-limited conditions, wild type (WT) strain grew better than Fur mutant strain. Furthermore, the fur mutant grew slower and had less terminal densities compared to the WT strain (23). In low iron conditions, the Fur mutant strain significantly lost flagella and decreased motility when compared with the WT. Furthermore, lower iron conditions induced rearrangements in energy metabolism, *C. difficile* significantly reduced the formation of most iron-requiring, ferredoxin-dependent processes. This adaptation was only partially regulated by Fur (23). The transition period for adaptation to low iron conditions represented a period of metabolic weakness and physical vulnerability, as demonstrated by the increased susceptibility to vancomycin and polymyxin B of the mutant (23).

Another study (24) investigated the Fur control putative iron acquisition systems *in vitro* and *in vivo* in *C. difficile* infection model. The Fur mutant strain grew normally in high and low-iron conditions when compared with WT. However, the Fur mutant exhibited low growth during the late stationary phase (24). The authors also observed that the Fur mutant present more than 70 upregulated and 44 downregulated transcripts compared with WT. 14 Fur-repressed genes, also were iron repressed. Interestingly, among those Fur- and iron-repressed genes were *C. difficile* genes encoding 7 putative cation transport systems. The expression of the flavodoxin gene, *fldX*, was suppressed by Fur and by high iron. In another way, the ferredoxin gene had lower expression either in mutant or under low-iron conditions (24). Moreover, Fur-regulated and iron-regulated genes increased *in vivo* during CDI using the hamster model. In general, Fur and iron were demonstrated to regulate several classes of ion transporters in *C. difficile* either *in vitro* or *in vivo* for regulating bacterial homeostasis (24).

Monaghan et al. (25) investigated the effects of the ferrous iron uptake system FeoB1 and the ferric uptake regulator and iron-dependent global gene regulator Fur, in a human *in vitro* gut model of C. difficile infection. The authors used two knockout mutants, an in single *feoB1* and other in *fur* homolog. Cytotoxicity assays revealed that the fur mutant strain produced considerably lower toxin levels than the feoB1 and WT strain (25). These results demonstrated the importance of the Fur system in regulating the expression of *C. difficile* toxins.

**Figure 2:**
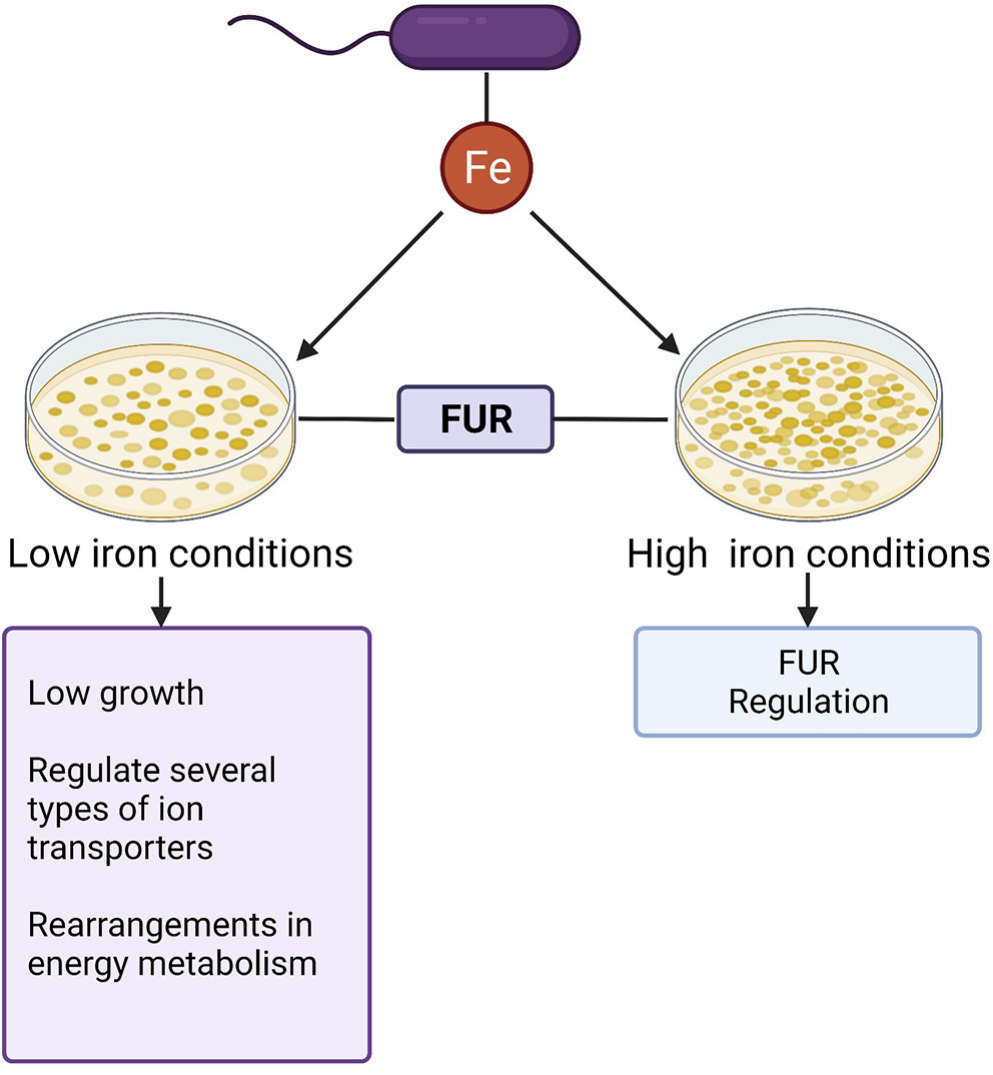
Role of iron in *C.difficile* homeostasis: Iron (Fe) represents a key cofactor for *C.difficile* homeostasis. The ferric uptake regulator (Fur) contributes to the control of intracellular homeostasis in both low high iron conditions.

Iron significantly influences the growth of *C. difficile* (26, 27). Current reports point out that from the first 5 h of culture, the presence of this mineral increases the growth of 360 strain, while in iron deprivation the growth rate is reduced followed by upregulation of genes linked to cellular iron uptake. Another important data concerning the growth of *C. difficile* shows that the addition of ferrous sulfate (FeSO4), iron chloride (FeCl3), iron citrate, and ferritin to the broth media in an iron-depleted environment allowed the bacteria to grow better when compared to media without iron supplementation (27). These findings suggest the importance of this micronutrient in *C. difficile* growth and the initiation of pathogenesis. Figure 2 summarizes the role of iron in *C. difficile* homeostasis.

In addition to the importance of iron for pathogen homeostasis, there appears to be a relationship between hemoglobin levels and CDI in humans (28, 29). Hemoglobin is the iron-containing pigment that enables red blood cells to carry high concentrations of oxygen to the tissues (30). A retrospective cohort study of 84 patients with CDI demonstrated that the hemoglobin levels were significantly low in patients with CDI recurrence (28). Another study carried out with 294 patients demonstrated that low hemoglobin levels (below 9 g/dL) may have prognostic significance, of colectomy or death in IBD patients with CDI (29).

Recent findings reported the connection between metronidazole resistance and dependence on undegraded heme stands out. The study by Wu et al. (31) noted that strains known to be resistant to metronidazole appeared more susceptible to the antibiotic when tested in heme-free media or in heme-containing media that were exposed to light (likely due to heme decomposition). The authors speculate that metronidazole-resistant *C. difficile* uses heme to recover in the presence of metronidazole. These results corroborate with a study conducted for Boekhoud et al. (32) that investigated resistance to metronidazole in a collection of clinical isolates of *C. difficile* and observed that heme supplementation from blood agar plates was the crucial determinant for four strains determined to be resistant to metronidazole. This effect would presumably be related to the ability of heme to detoxify the nitro-radicals generated by metronidazole activation.

### Zinc

Zinc (Zn) is an essential micronutrient acquired through the diet and is recommended by the World Health Organization (WHO) in cases of acute diarrhea (33). CDI is often associated with nosocomial diarrhea, and zinc deficiency appears to be related to the recurrence of CDI (33).

Three papers have implicated the importance of zinc in patients with CDI: a case study (34) and two retrospective cohort studies (33, 35), The case report showed the history of a 52-year-old diabetic patient who had undergone kidney transplantation and developed recurrent CDI, presenting with persistent diarrhea and weight loss of 13kg. The initial treatment was with vancomycin. After the third recurrence of CDI, an evaluation of serum Zn was carried out, and low serum zinc level (36 μg/dL) was found. The patient underwent another cycle of vancomycin, however, in combination with zinc supplementation. Improvement of symptoms, including diarrhea, without a new CDI recurrence episode, was reported (34).

In a retrospective cohort study of 113 patients, zinc was the most common micronutrient deficiency in patients with CDI, and it was observed that Zn supplementation may decrease the association between zinc deficiency and recurrent CDI (35). Similarly, in another retrospective cohort study of 127 patients, zinc deficiency was associated with higher rates of CDI recurrence after fecal microbiota (FMT) transplantation. Zinc supplementation in zinc-deficient patients, associated with reduced recurrence rate in these patients (33).

In contrast, a study using a CDI model in mice showed that a diet rich in zinc (1,000 mg/kg) increased the toxin's activity, altered the immune response (increased IL-1β and decreased levels of IL-6 and IL-12 cytokines and anti-inflammatory cytokine IL-10), and increased the severity of the disease (36). In this same study, deficiency of calprotectin (CP), which is a calcium and zinc binding protein, did not alter the stool shedding of *C. difficile* in mice (36). Another study demonstrated a putative zinc transporter (ZupT) is used by *C. difficile* as a mechanism for survival due to high CP levels-mediated decreases in availability of Zn (37).

In another mouse model of CDI, CP limited the availability of Zn and increased the transcription of *C. difficile* genes necessary for proline fermentation. This reduced fermentation is due to the limited availability of another nutrient needed for proline fermentation, selenium. Therefore, CP-mediated Zn limitation combined with selenium deficiency affects the proline fermentation necessary for broader adaptation of *C. difficile* in the host gut (38).

**Figure 3:**
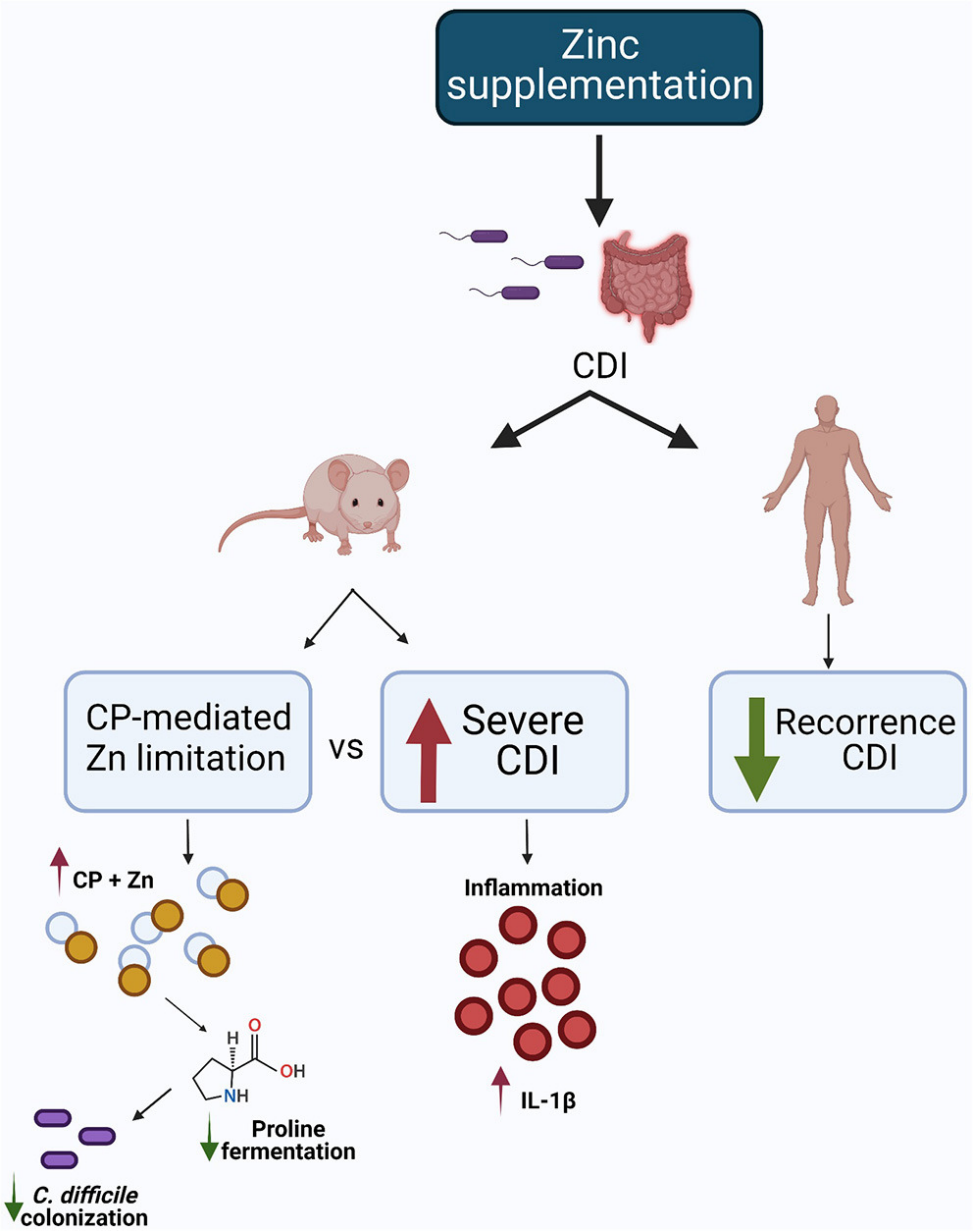
Possible mechanism of zinc in CDI. In the animal model, one study showed that dietary Zn supplementation increased CDI severity, while another demonstrated calprotectin-mediated zinc limitation, reducing proline fermentation and, consequently, *C. difficile* colonization. In the human study, Zn supplementation reduced CDI recurrence. Created with BioRender.com.

These inconsistent effects of zinc in *C. difficile* infection may be related to supraphysiologic levels of zinc in the mouse studies. The Figure 3 summarizes the possible mechanism of zinc in CDI.

### Vitamin D

Vitamin D is an essential dietary nutrient known for its critical function in bone homeostasis. It is also involved in absorption of inorganic elements (such as calcium, phosphorus, and magnesium), kidney dysfunction (by reducing proteinuria and inflammation) and immune responses (39–42). There are two forms of vitamin D, ergocalciferol (vitamin D2), and cholecalciferol (vitamin D3). Vitamin D deficiency increases the risk of autoimmune disease and allergies (43), as well as causes alterations in intestinal barrier and changes in microbiome composition (44). Moreover, some studies have demonstrated a strong correlation between vitamin D deficiency and incidence/severity of some infectious diseases such as respiratory infection, tuberculosis, and CDI (45–47).

In patients with CDI, it was demonstrated that there is a significant association between low vitamin D 25 (OH) D3 levels and CDI severity (48). Accordingly, patients with normal levels of vitamin D (>20 ng/mL) presented with non-severe CDI, although the low level of vitamin D was not associated directly with increased risk of developing CDI and mortality in hospitalized patients. The level of vitamin D did not correlate with CDI recurrence (49).

In a study with 271 patients with CDI, 17.5 % of patients with severe CDI had deficiency of Vitamin D [25 (OH) D <10 ng/mL] (50). Additionally, Gayam et al. (51) and Kanamori et al. (52) found that patients with CDI and vitamin D deficiency (<10 ng/mL) are more susceptible to developing sepsis and peritonitis, resulting in increased medical treatment costs and mortality for this group of patients. Ananthakrishnan et al. (53) and Sahay et al. (54) also demonstrated that low levels of vitamin D (<20 ng/mL) were associated with increased risk of CDI in patients with inflammatory bowel disease (IBD) while higher plasma calcifediol [25 (OH) D] was associated with reduced risk. A negative correlation between vitamin D levels and the risk of developing community-acquired *C. difficile* infection (CA-CDI) has been reported (55). In addition, low vitamin D levels and age > 70 years old were independently associated with increased recurrence of *C. difficile* associated diarrhea (56). Consistent with these findings, CA-CDI cases was previously associated with low serum levels of 25 (OH) D (>15 ng/mL) measured up to 12 months or 2 weeks after toxin positivity (54).

Drall et al. (57) showed that maternal consumption of vitamin D-fortified milk reduced the *C. difficile* colonization in infants by a microbiota independent mechanism, as no difference was found in microbiota composition of infants breastfed with vitamin D-fortified milk and not breastfed. In contrast, Singh et al. (58) showed that vitamin D supplementation increased gut microbial diversity and specifically increase the abundance of *Bifidobacterium* which has been related to reduce manifestation of CDI (59, 60). The microbial diversity is essential to reduce the susceptibility to intestinal infections (61).

Impairment of innate and adaptative immune responses during infections are increasingly being related to hypovitaminosis D (62). This is because vitamin D has an important effect on clearance of pathogenic organisms by stimulating the production of cathelicidin, an antimicrobial peptide that has also shown to inhibit the effect of *C. difficile* toxins (63). Cathelicidin is a key antimicrobial peptide in mammals, being found in sites of inflammation and is involved in the host defense against bacteria and other pathogens (64).

**Figure 4:**
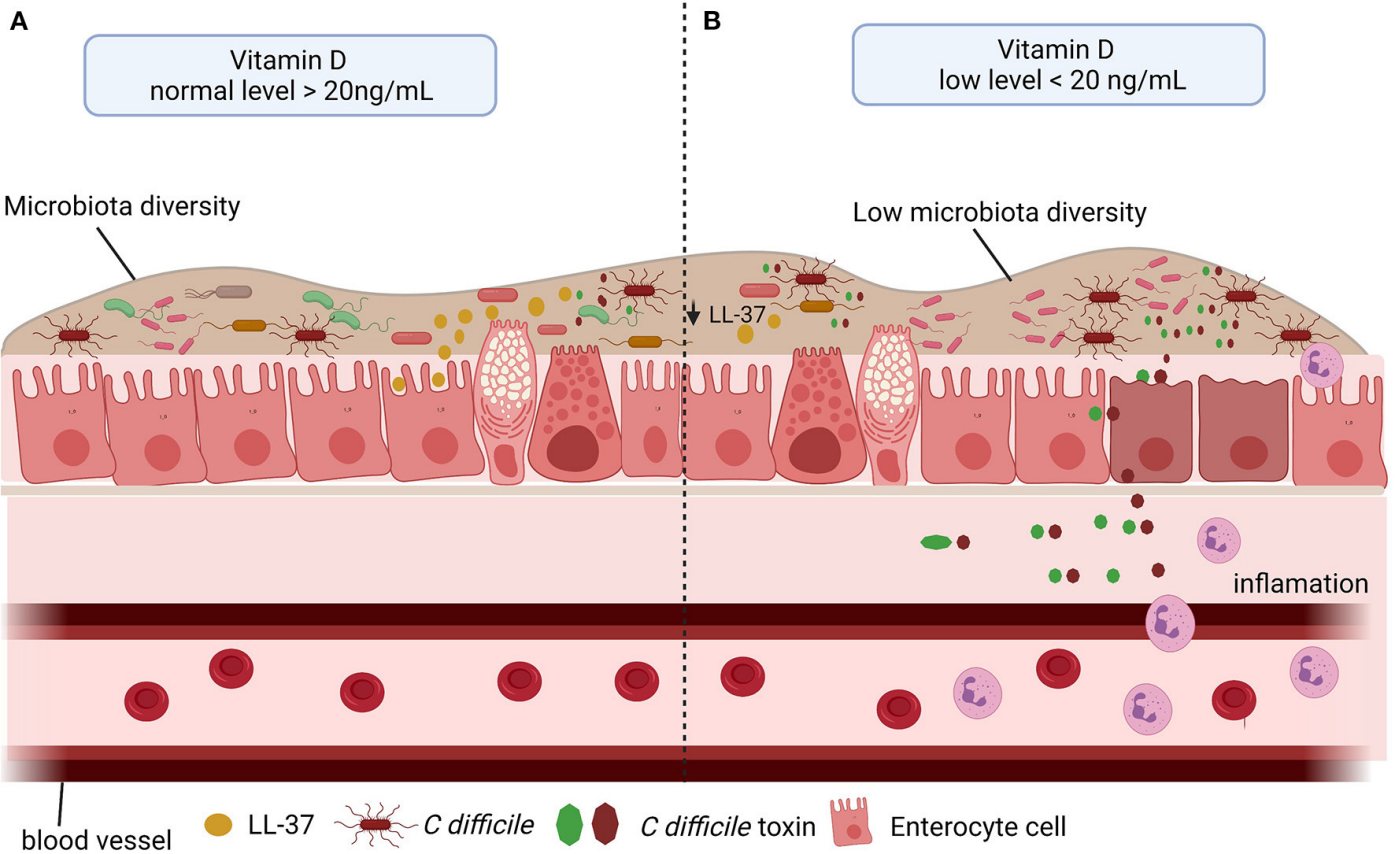
Vitamin D level and *C. difficile* infection (CDI). **(A)** The vitamin D stimulates the microbiota diversity and the cathelicidin (LL-37) production, which in turn inhibits the effect of *C. difficile* toxins. **(B)** Low level of vitamin D impacts negatively microbiota diversity and in LL-37 production, consequently, promotes the *C. difficile* colonization, toxin production and inflammation.

Taken together all these findings, vitamin D deficiency may be associated with an increased risk of CDI by reducing cathelicidin production, resulting in decreased intestinal microbiota diversity. Thus, vitamin D plays an important protective role during CDI. The Figure 4 summarizes the role of vitamin D in CDI.

### Glutamine

Glutamine (L-Gln) is a nonessential amino acid (NEAA) due to the endogenous glutamine biosynthesis pathway. L-alanyl-L-glutamine (AQ) is a stable source of L-Gln used in clinical and pre-clinical studies. L-Gln plays an important role in tricarboxylic acid (TCA) cycle supplementation and in biosynthesis of nucleotides, glutathione (GSH), and other nonessential amino acids. The L-Gln transporters (SLC1A5, SLC38A1, and SLC38A2) can mediate the L-Gln transportation into cells through plasma membrane, once in the cytoplasm, it can be used to the biosynthesis of hexosamine, nucleotides, and asparagine (65).

*In vitro*, L-Gln protected intestinal epithelial cells (IEC-6) from the deleterious effects of TcdA by promoting proliferation and inhibiting apoptosis via increasing RhoA expression (66). RhoA is a Rho GTPase that has a key inductor role in proliferation and migration, its deletion decreases these functions in other cells (67). Similarly, L-Gln also protected rat intestinal epithelial cells (IEC-6) challenged with TcdA and TcdB by increasing proliferation and migration, and by decreasing apoptosis (68–70). Glutamine is also important to the host response and its depletion potentiates leucocyte-dependent inflammatory events induced by TcdA in rats (71). In rabbit ileum and mouse cecal tissues challenged with TcdA, AQ potentialized the effect of A2A agonist in decreasing apoptosis, intestinal damage and proinflammatory cytokine secretion (72). On the other hand, in a model of CDI, AQ decreased intestinal damage only when mice were treated with vancomycin (68). These data suggest that the AQ-related protective effects do not occur by directly targeting *C. difficile* during infection.

**Figure 5:**
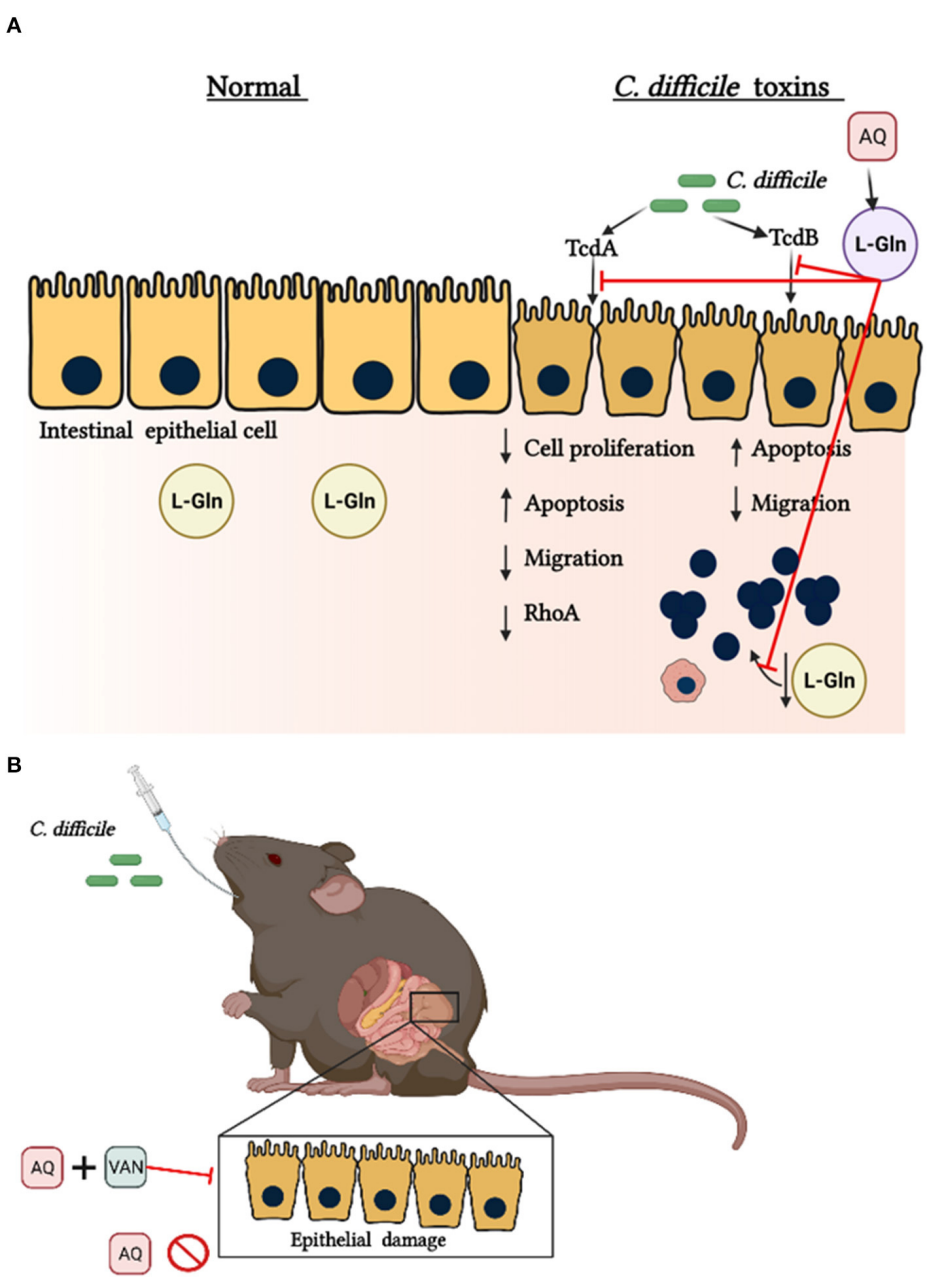
Protective effects of glutamine during *C. difficile* toxins Challenge. **(A)** In normal conditions endogenous glutamine (L-Gln) maintains epithelial functions. *C. difficile* toxin A (TcdA) and *C. difficile* toxin B (TcdB) promote a decrease in intestinal epithelial cell proliferation, migration and RhoA levels. *C. difficile* toxins also decrease the levels of L-Gln into the intestine. Alanyl-glutamine (AQ), as a source of L-Gln, inhibits these toxins' effects. **(B)** AQ decreases the intestinal epithelial damage during CDI only if infected mice receive vancomycin (VAN).

Further, in a metabolomics analysis of cecum content after 30 h post-CDI, levels of glutamine showed to be elevated (73). However, because of its short half-life, glutamine may not be able to stimulate host cells such as epithelial cells to generate its protective effects during infection. More studies to better understand the mechanism involved in the beneficial outcome promoted by AQ supplementation during CDI are still needed. The Figure 5 summarizes the possible mechanism of glutamine in *C. difficile* toxins challenge.

## Conclusion

Here, we showed that nutrients play an important role in modulating host and pathogen response during CDI. Calcium facilitates spore germination and TcdB-induced ROS synthesis. Selenium reduces the cytotoxicity of *C. difficile* toxins by decreasing the production of these toxins in *C. difficile*. Vitamin D deficiency is strongly associated to CDI severity, once vitamin D plays an important role in stimulating cathelicidin synthesis, which in turn inhibits the toxins' effects. Whereas the beneficial effects of glutamine in CDI seems to be by modulating the host response. However, the beneficial effects of Iron and Zinc during CDI appears to be level dependent. However, further studies are needed to better characterize the role of nutrients in the interaction between the host components and *C. difficile* and clarify some controversial results. We believe it is important to investigate nutritional deficiencies during CDI in human.

## Author Contributions

AL, MB, MM, IS, LT, CM, and DC wrote sections of the manuscript. AL, MB, AS, RL, JS, CW, DC, and GB contributed to the organization of the manuscript, reviewed, and approved the final version. All authors contributed to the article and approved the submitted version.

## Funding

This work was supported by CAPES and CNPq of Brazil (PRONEX CNPq/FUNCAP grant number PR2-0101-00060.01.00/15).

## Conflict of Interest

The authors declare that the research was conducted in the absence of any commercial or financial relationships that could be construed as a potential conflict of interest.

## Publisher's Note

All claims expressed in this article are solely those of the authors and do not necessarily represent those of their affiliated organizations, or those of the publisher, the editors and the reviewers. Any product that may be evaluated in this article, or claim that may be made by its manufacturer, is not guaranteed or endorsed by the publisher.
